# Novel linkage approach to join community-acquired and national data

**DOI:** 10.1186/s12874-024-02143-3

**Published:** 2024-01-17

**Authors:** Claire Tochel, Emma Pead, Alice McTrusty, Fiona Buckmaster, Tom MacGillivray, Andrew J. Tatham, Niall C. Strang, Baljean Dhillon, Miguel O. Bernabeu

**Affiliations:** 1 Centre for Medical Informatics, University of Edinburgh, Edinburgh, UK; 2 Centre for Clinical Brain Sciences, University of Edinburgh, Edinburgh, UK; 3 Princess Alexandra Eye Pavilion, NHS Lothian, Edinburgh, UK; 4 Department of Vision Sciences, Glasgow Caledonian University, Glasgow, UK

**Keywords:** Community optometry, Data linkage, Early disease detection, Longitudinal data, Image analysis

## Abstract

**Background:**

Community optometrists in Scotland have performed regular free-at-point-of-care eye examinations for all, for over 15 years. Eye examinations include retinal imaging but image storage is fragmented and they are not used for research. The Scottish Collaborative Optometry-Ophthalmology Network e-research project aimed to collect these images and create a repository linked to routinely collected healthcare data, supporting the development of pre-symptomatic diagnostic tools.

**Methods:**

As the image record was usually separate from the patient record and contained minimal patient information, we developed an efficient matching algorithm using a combination of deterministic and probabilistic steps which minimised the risk of false positives, to facilitate national health record linkage. We visited two practices and assessed the data contained in their image device and Practice Management Systems. Practice activities were explored to understand the context of data collection processes. Iteratively, we tested a series of matching rules which captured a high proportion of true positive records compared to manual matches. The approach was validated by testing manual matching against automated steps in three further practices.

**Results:**

A sequence of deterministic rules successfully matched 95% of records in the three test practices compared to manual matching. Adding two probabilistic rules to the algorithm successfully matched 99% of records.

**Conclusions:**

The potential value of community-acquired retinal images can be harnessed only if they are linked to centrally-held healthcare care data. Despite the lack of interoperability between systems within optometry practices and inconsistent use of unique identifiers, data linkage is possible using robust, almost entirely automated processes.

## Introduction

Retinal images, either from fundus photography or from imaging devices such as optical coherence tomography provide valuable insights into ophthalmic and systematic diseases. This has the potential to improve the ability to detect sight or life-threatening conditions at an earlier stage. Development of robustly labelled, real-world datasets is essential for obtaining the large number of images need to develop and validate tools for disease detection and risk stratification, however, to date the majority of such datasets have been derived from images obtained in secondary care and therefore may not be generalisable if tools are to be used in primary care or population-based settings.

There is widespread public support for the use of linked, routinely collected data to support health research [1]. This support is conditional on perceived public benefit and concerns about potential harms. Awareness about existing practice around data linkage is reported to be low. The need for ‘adaptive governance’ which remains responsive to both researcher and public needs and expectations (which may change over time) has been highlighted as a key element in maintaining public acceptability in this field [2].

Scotland has several decades of health data linkage between hospital discharges, cancer registrations, medications and deaths [3–5]. The process of robustly linking electronic records benefited from the development in the 1970s of a national unique identifier (Community Health Index (CHI)), initially applied to primary care data [6]. Multiple National Health Service (NHS) healthcare datasets are now routinely collated centrally by Public Health Scotland and populated with CHI [7–9]. Accurate and efficient linkage of information for the same individual from different sources, is fundamental to allow meaningful and up to date research.

Scotland has a unique model of primary care eye examinations which, since 2006, have been funded by the NHS, removing the cost barrier to accessing eye care. Eye examinations are conducted by community optometrists and involve a thorough assessment of eye health, including fundus examination, visual field assessment, and refractive error. Retinal photography became standard for all patients over 60 years of age in 2008 [10]. This means that many community optometry practices have been capturing and storing retinal images for well over a decade and nationally optometrists now take millions of retinal images every year. The large number of retinal photographs obtained across the older population including both healthy and diseased individuals provides a valuable potential resource for longitudinal analysis, particularly as images acquired in primary care as part of the routine eye examination are likely to make the dataset more representative of the population in which screening or early diagnostic tools would be employed.

The Scottish Collaborative Optometry-Ophthalmology Network e-research (SCONe) project was set up [11] as a result of a shared vision among clinicians, researchers and patient-supporting charities, that these retinal images could yield enormous benefit beyond the delivery of individual health care [12–14]. The SCONe resource would be a valuable addition to the current array of ophthalmic datasets many of which are created with a bias towards inclusion of cases with one particular disease [15]. To achieve this, SCONe is retrieving retinal images captured routinely at community optometry practices and linking them to NHS data within the Scottish National Safe Haven (NSH), creating a longitudinal research resource to support development of new technologies for early detection of eye disease, risk prediction, and discovery of retinal biomarkers of body and brain health [13, 14]. The NSH provides a secure technical and governance framework for linked data projects including secure access to NHS data, and ethical approval for research conducted there (https://www.ed.ac.uk/edinburgh-international-data-facility/services/safe-haven-services/scottish-national-safe-haven).

The benefits and risks of bringing separate datasets together to support health research are well documented [16, 17]. Ideally a unique and common identifier is available [18, 19]. Without this, it is necessary to develop a bespoke approach to matching records using robust techniques which must be guided by features in the data. Probabilistic (which calculate the likelihood of a match comparison) and deterministic (which result in a binary yes/no result) matching methods potentially bring together records with different degrees of efficiency, sensitivity and specificity [20–22]. For this work to be done at scale, manual intervention must be minimised, without introducing false matches.

Most optometry data are collected in busy patient environments and in systems which may not be interoperable with each other. Whereas these data meet the requirements of the practice to provide healthcare services, they may not meet the stringent quality requirements for research through linkage to other healthcare data, such as the routine inclusion of CHI. The SCONe Proof of Concept study aim was to test the technical feasibility of exporting colour fundus photographs for patients aged 60 and over from practice camera devices, with enough patient information to facilitate linkage to national hospital and ophthalmic data within the NSH [23].

The objectives of this study were to develop a novel automated linkage process which accurately identified PMS records for patients with retinal images captured in community optometry practices which are commonly identified only by forename, surname and date of birth, and validate this against manual linkage.

## Methods

Following SCONe’s early engagement (surveys, presentations, newsletters, social and mainstream media articles) with community optometry across Scotland in 2020, some highly motivated practitioners (optometrists and practice directors) came forward to volunteer as pilot practices for the Proof of Concept study. The NHS Scotland Public Benefit and Privacy Panel for Health and Social Care approved the study in 2021. The NSH is the host of an existing imaging project which hosts radiological scans for research, and SCONe is working with the same team [24, 25].

A data sharing agreement was set up between each pilot practice’s data controller and the study’s co-sponsors (University of Edinburgh and NHS Lothian) and the authors arranged site visits to each practice in turn. Practices provided details of the hardware and software used by the practice to store retinal images and patient data. Test exports were run within practice in advance where possible to establish whether password access would be necessary at any point in the process, gauge the time required to carry out each tasks, verify that outputs would be in a usable format and explore any additional technical input which might be necessary. Additional information was sought from device manufacturers.

For robust linkage to national data to be performed within the NSH, six fields are required: patient forename, surname, date of birth (DOB), sex, address, and postcode. In optometry practices the Practice Management System (PMS) is used to register new patients, manage bookings and store clinical information; PMS records typically contains all six required linkage fields. Retinal images captured in practice are often stored in structured query language databases and the associated patient records tend to contain only the patient’s forename, surname, and DOB. On site, PMS data were exported to a spreadsheet and images were exported from the databases as jpeg, tiff or png images with an associated extensible markup language file containing image filename, forename, surname, and DOB.

### Data preparation

Patient forename and surname from the image device and PMS were first cleaned by converting all characters to lower case and removing non-letter characters or spaces using R v4.1.3 [26]. Match comparisons were created using “phonics” and “stringdist” packages.

### Developing “ground truth” for match comparison

Within each of two test practices, an inner join operation was applied between image and PMS data, with perfect matches identified and removed from subsequent matching steps. Assessment of the PMS data and imaging records which did not match, revealed many potential missed matches due to slight differences. These appeared to include both intentional and unintentional differences with apparent ‘errors’. Perceived intentional differences included truncation or expansion of names and unintentional ones included incorrect dates of birth, spelling errors, missing or extra spaces, hyphens and apostrophes. For the purpose of matching these were all considered ‘errors’ in patient information. They were more prevalent on the camera device exports compared to the more complete PMS records.

Review and discussion of these cases by the authors, supplemented by professional experience in optometry practices led to a consensus around the extent of error which should be tolerated and therefore which potential matches to include and reject. To create optimal matching rules for the algorithm, several different deterministic and probabilistic matching techniques were explored iteratively. We evaluated the range of acceptable typographical errors and alternative names identified without inadvertently linking records erroneously. Based on the literature, deterministic rules were considered preferable to probabilistic rules to minimise the need for manual review of scores in the algorithm [22].

A list of manual matches was created for each practice (i.e. the “ground truth”) based on individual review of camera device records which had not matched to a PMS patient record, but for which the author perceived that the mismatch fitted within the agreed rules and that the mismatch was likely due to an error. Whereas this approach identified many more linkable images, it was very time consuming (approximately three person-days for a single practice with 2,000 unmatched images) and included the risk of human error.

Based on the information gathered from the first two practices, we developed a sequential set of rules to match patients from the image device data to the PMS patient list with a high true positive rate and low false positive rate compared to the manual matching process. We tested this same sequence in three further practices.

**Fig. 1:**
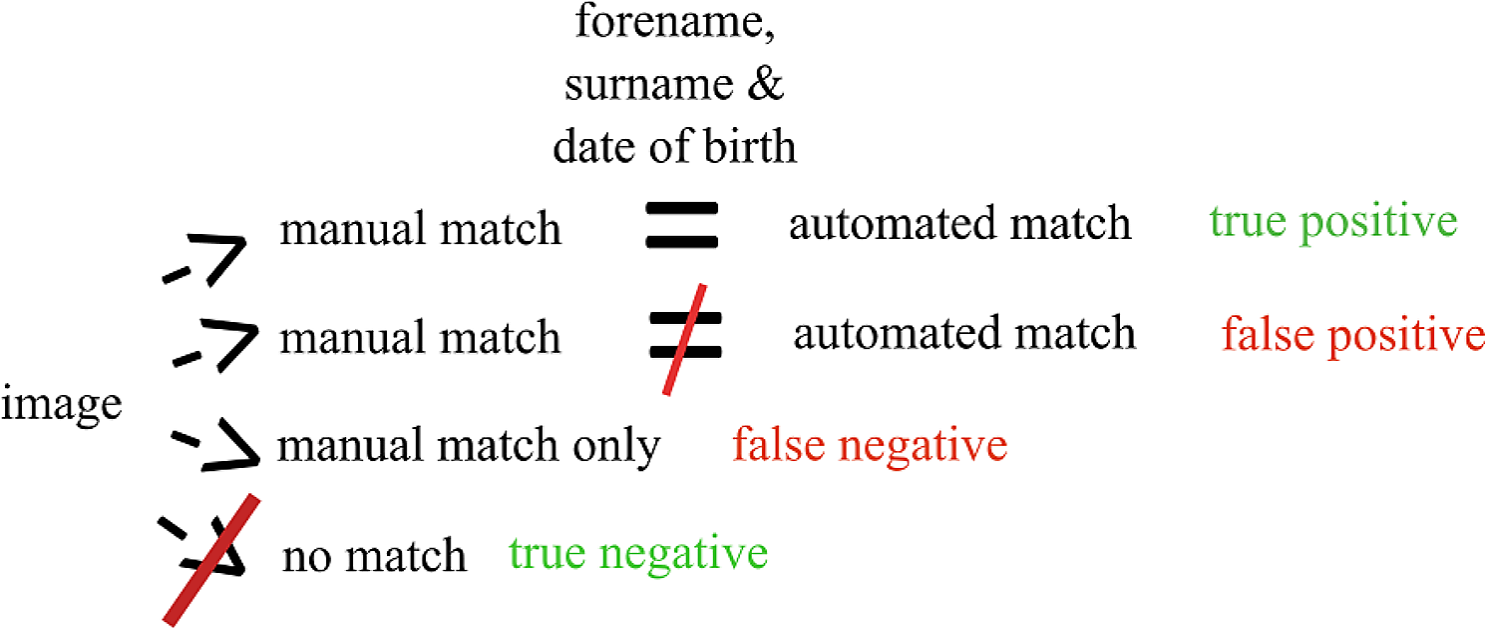
Schematic showing definition of the four potential outcome matches for an image comparing manual and automated matching processes

The following definitions were used to calculate true and false positive rates when comparing the matching done via the automated sequence to the manual process (Fig. 1):

- true positive: image was associated with the same individual in the automated and manual match lists
- false positive: image was associated with a different individual in automated and manual match lists
- false negative: image was not associated with an individual in automated list but was in manual list
- true negative: image was not associated with an individual in automated or manual match lists.

## Results

### Development of automated matching sequence

The most useful techniques for matching names were selected based on iterative exploration of the early pilot practices. Variations such as Muhammad and Mohammed were matched by converting each string to a Soundex code (M530) based on a phonetic comparison [26]. Truncations or expansions (e.g., “Elizabeth” to “Eliza”) could be detected using the Longest Common Substring (LCS) calculation [27]. This is based on the longest string that can be obtained by pairing characters in the two names while keeping the order of characters intact. The LCS distance is calculated from the number of unpaired characters meaning that low scores usually indicate stronger matches.

Other ‘edit distance’ calculations comparing names such as Levenshtein and Jaro-Winkler distance did not improve detection of matches [27]. The Jaro-Winkler distance (whereby a perfect match between two strings gives a score of one) in some cases returned a low score for what appeared to be clear matches during manual review. The Levenshtein distance (where zero indicates a perfect match, but the maximum score depends on the number of characters in the longer of the two strings) performed similarly to LCS, but as the latter was a simpler measure on which to apply a cut-off given the observed naming errors, it was used. A score of 6 was found to be a suitable threshold.

With respect to DOB, after review of commonly occurring errors, it was decided that an error in one number (day, month, year) or the reversal of day and month was acceptable in the presence of other matching details.

### Validation

In the three validation practices, the first step (identical forename, surname and DOB) matched between 84% and 91% of images to a patient in the PMS. These cases were not further scrutinised. Manual matching typically added a further 8-15% of images successfully linked to a PMS patient record.

The success of each automated matching step compared to the manual match list in each practice was calculated, with those matches removed from subsequent steps (Table 1).

1. Exact forename, surname and DOB
2. Exact forename and surname, DOB error tolerated
3. Exact DOB, Soundex forename and surname
4. Soundex match on forename and surname with DOB error tolerated
5. Exact DOB and surname, LCS forename under threshold (6 used in these tests)
6. Exact DOB and forename, LCS surname under threshold (6 used in these tests).

All of the false positive matches identified during the final two stages in practice 1 were manually reviewed, and were deemed to be true matches, but for which information had been manually corrected in the PMS data to fill missing details from another source. By eliminating this process from the 2nd and 3rd practices, no false positives were created using the automated linkage steps.

### Beyond the pilot

**Fig. 2:**
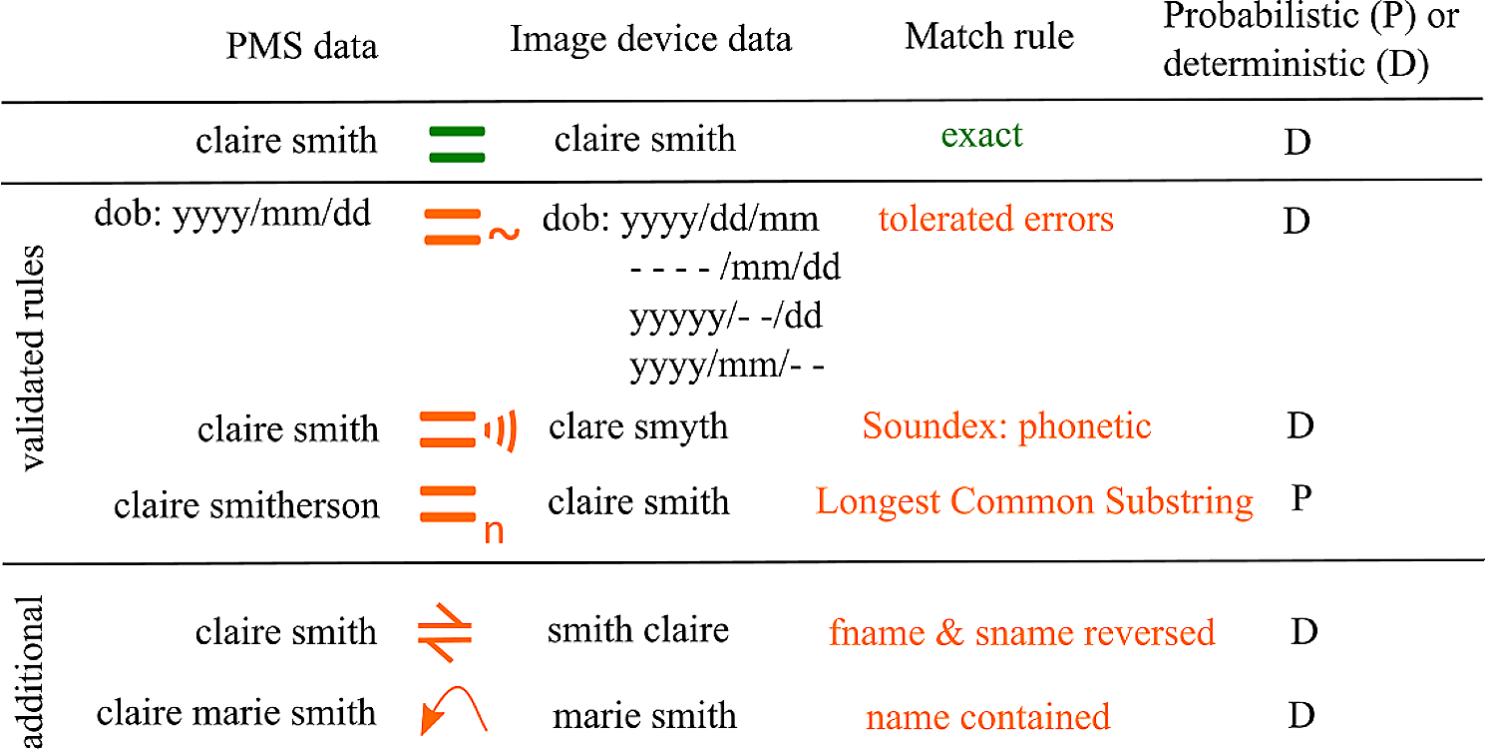
Schematic of deterministic (D) and probabilistic (P) rules used in the matching algorithm. Green indicates an exact match, amber indicates that the match between the image and Patient Management System (PMS) record met a pre-defined rule. Date of birth (dob) is based on year (yyyy), month (mm) and day (dd)

Work on SCONe has continued with seven more practices visited at the time of writing. Further deterministic steps have been found to be necessary to catch obvious potential matches where new local anomalies in data entry were evident after the final step described above. For example, some practices include middle names with forename and double-barreled surnames in the PMS but not the image record. The additional steps added (but not validated with a full manual check) were: forename from image list contained entirely within PMS forename; surname from image list contained entirely within PMS surname and forename and surname reversed in image list. To minimise the two probabilistic matching steps (and therefore the number of matches requiring manual review) these three steps have been added before using LCS. In subsequent practices the ranked match scores have been reviewed, and the score of 6 remains a useful threshold to minimise false positives, however occasionally obvious outliers can be matched by making a manual intervention. All useful steps identified to date are shown schematically in Fig. 2.

**Table 1:** Number of true and false matches made between image and Patient Management System records within each practice by each rule when compared to manual matching after step 1. True positive and false positive rates provided in brackets

Practice (number of patients)	Match step	True positive (rate)	True negative	False positive (rate)	False negative	Number of images
**Practice #1 (2,339)**						
	1	18,699 (0.859)	4,890	0 (0)	3,079	26,668
	2	19,338 (0.888)	4,881	0 (0)	2,449	26,668
	3	20,696 (0.95)	4,881	0 (0)	1,091	26,668
	4	20,735 (0.951)	4,866	0 (0)	1,067	26,668
	5	21,495 (0.986)	4,823	43 (0.009)	307	26,668
	6	21,547 (0.988)	4,788	78 (0.016)	255	26,668
**Practice #2 (309)**						
	1	1,003 (0.836)	803	0 (0)	197	2,003
	2	1,043 (0.869)	803	0 (0)	157	2,003
	3	1,133 (0.944)	803	0 (0)	67	2,003
	4	1,133 (0.944)	803	0 (0)	67	2,003
	5	1,155 (0.963)	803	0 (0)	45	2,003
	6	1,185 (0.988)	803	0 (0)	15	2,003
**Practice #3 (133)**						
	1	247 (0.911)	5	0 (0)	24	276
	2	249 (0.919)	5	0 (0)	22	276
	3	263 (0.97)	5	0 (0)	8	276
	4	263 (0.97)	5	0 (0)	8	276
	5	266 (0.982)	5	0 (0)	5	276
	6	269 (0.993)	5	0 (0)	2	276

Match steps:

1. Exact forename, surname and DOB

2. Exact forename and surname, DOB error tolerated

3. Exact DOB, Soundex forename and surname

4. Soundex match on forename and surname with DOB error tolerated

5. Exact DOB and surname, LCS forename < 6

6. Exact DOB and forename, LCS surname < 6

True positive rate = true positive / (true positive + false negative)

False positive rate = false positive / (false positive + true negative)

## Discussion

We have developed an algorithmically-defined matching sequence with deterministic and probabilistic rules which, applied to limited data (three identifying fields for each image), facilitated rapid matching to a more detailed patient record, on a different and unconnected system with high true and low false positive rates. The first step (perfect match of three fields) yielded at least an 84% match and application of the rules increased this to 99% with no false positives compared to ground truth. These rules were selected, based on a thorough review of the datasets in multiple practices, considering the known behavioural and technological factors at play in practice to assess the likelihood of error. Assuming that the manual match is adequately accurate, these examples suggest that the algorithm rules can deliver a robust and efficient matching process for future practices including those with very large patient lists. The process continues to be developed and refined in response to new experiences in practice.

### Real world data challenges

The lack of interoperability between image capture devices and PMS in most community optometry practice is challenging on multiple levels. It means that staff must manually enter patient details in order to take a retinal image, despite the full patient record already existing just metres away. This is inefficient and inevitably leads to the potential for error. From a research perspective, it makes the data harder to incorporate into healthcare datasets, as these must be linked to the patients’ unique identifier which needs accurate and detailed patient information.

The Scottish Government’s Digital health and care strategy, refreshed in 2021, emphasised the need for data to be used to benefit the country’s citizens, and acknowledged that people are frustrated at the lack of co-ordination and links between different parts of the healthcare system [28]. This work, and the SCONe project generally, is an example of the innovation required to securely bring together elements of that system which are currently fragmented, to harness benefits which are impossible with data stored in isolation.

Patients whose images were not matched to the full PMS record, risk not being matched to their unique identifier (CHI) within the NSH and will therefore be missing from the linked research resource. If these individuals are lost at random from practice lists, then this will not be problematic other than the loss of beneficial data to any work done on the images. However, if there is a systematic reason behind the lack of matches, the cohort may not be representative of the community. For example we may disproportionately lose married women who have changed their surname (in systems where this is not retained in their record), or people from ethnic groups among which names don’t neatly fit into systems with a single forename and surname format. If an image is incorrectly matched to the wrong CHI, the wrong individual’s health records will be included in the linked dataset. Clearly this has the potential to lead to inaccurate research findings which would undermine the project, so methods for quality assessment need to be built in [16, 29]. It also poses a problem from the governance perspective, in that the project has permission to use records from a defined subset of the population. Both adverse implications must be avoided at all costs, and we have therefore taken a strict approach whereby the risk of losing true matches is preferable to including false matches.

### Limitations

The risk of matching a patient’s image with a different patient’s record within each practice based on the three available fields, was considered to be much lower than a similar matching process conducted nationally (for which six fields are considered necessary). The relatively (and in some cases absolutely) small number of patients and localised geographical area from which each practice population was drawn meant that this novel approach with the agreed error tolerance was considered acceptable to the authors. All practices involved in this exercise were independently owned with small numbers of staff inputting information into the image device and PMS. Different rules and error tolerance may be required if matching involved larger practices with more staff and more potential variation in data input. The data collection and systems are also likely to vary between practices meaning that there is likely no single set of rules which would apply optimally in every practice. However, these rules, based on a logical sequence of low risk measures, worked in practices with very different size of patient list (20,000 vs. 27,000) and we have continued to develop the process as we visit more sites. We currently do not have a method to verify image matching, i.e. does any given retinal image actually belong to the named patient.

## Conclusion

To date the SCONe team have delivered retinal images, captured over many years, for 4,000 patients to NSH, where they were matched to CHI. This study describes the necessary intermediate step of linking retinal images stored with just three personal data fields, to the six fields required for CHI linkage, which facilitated the creation of a cohort of 28,947 community-acquired images linked to routinely-collected healthcare data within the NSH for the first time. Evaluating the images linked to NHS data will allow us to assess the potential they contain, and work towards establishing a rich, longitudinal retinal image repository, which could grow year on year with the full support of Scottish Government [30]. This would realise SCONe’s potential to support the detection of pre-symptomatic disease and the development of improved diagnostic tools and treatments, directly benefitting the public whose images are its defining and most valuable feature.

## Data Availability

The datasets analysed during the current study are not publicly available because they were acquired from community optometrists who authorised their use through Data Sharing Agreements (once processed into the necessary specification) only within the National Safe Haven. After delivery to the National Safe Haven the images and linking data were deleted from University of Edinburgh systems as agreed in the permissions for the study. In the future we hope that the SCONe dataset (retinal images linked to pseudonymised healthcare data) will be available for use by researchers via the Public Health Scotland National Safe Haven. We are still in the early stages of developing this resource. The corresponding author is happy to prepare an anonymised version of the scripts used in the data processing to share on request.
